# *YELLOW LEAF AND DWARF 7,* Encoding a Novel Ankyrin Domain-Containing Protein, Affects Chloroplast Development in Rice

**DOI:** 10.3390/genes15101267

**Published:** 2024-09-27

**Authors:** Yongtao Cui, Jian Song, Liqun Tang, Jianjun Wang

**Affiliations:** Institute of Crops and Nuclear Technology Utilization, Zhejiang Academy of Agricultural Sciences, Hangzhou 310021, China; song521125@163.com (J.S.); liquntang2013@126.com (L.T.); wangjj4197@163.com (J.W.)

**Keywords:** rice, *YLD7*, grana, yellow and lesion-like leaf, chloroplast development

## Abstract

**Background:** The proper development of grana and stroma within chloroplasts is critical for plant vitality and crop yield in rice and other cereals. While the molecular mechanisms underpinning these processes are known, the genetic networks governing them require further exploration. **Methods and Results:** In this study, we characterize a novel rice mutant termed *yellow leaf and dwarf 7* (*yld7*), which presents with yellow, lesion-like leaves and a dwarf growth habit. The *yld7* mutant shows reduced photosynthetic activity, lower chlorophyll content, and abnormal chloroplast structure. Transmission electron microscopy (TEM) analysis revealed defective grana stacking in *yld7* chloroplasts. Additionally, *yld7* plants accumulate high levels of hydrogen peroxide (H_2_O_2_) and exhibit an up-regulation of senescence-associated genes, leading to accelerated cell death. Map-based cloning identified a C-to-T mutation in the LOC_Os07g33660 gene, encoding the YLD7 protein, which is a novel ankyrin domain-containing protein localized to the chloroplast. Immunoblot analysis of four LHCI proteins indicated that the YLD7 protein plays an important role in the normal biogenesis of chloroplast stroma and grana, directly affecting leaf senescence and overall plant stature. **Conclusions:** This study emphasizes the significance of *YLD7* in the intricate molecular mechanisms that regulate the structural integrity of chloroplasts and the senescence of leaves, thus providing valuable implications for the enhancement of rice breeding strategies and cultivation.

## 1. Introduction

Leaves are crucial for photosynthesis in green plants as they generate vital nutrients necessary for growth and development [1]. However, abnormal aging processes, such as leaf yellowing, can substantially diminish photosynthetic efficiency and carbohydrate accumulation, leading to reduced crop yields [2,3,4]. Genetically regulated and often environmentally stimulated, senescence plays a pivotal role in nutrient recycling that is essential for new growth [5,6,7,8,9]. Therefore, a comprehensive understanding of the molecular mechanisms controlling leaf senescence is pivotal for advancements in rice breeding and production.

Chloroplasts are integral to leaf cell metabolism in higher plants, comprising the chloroplast envelope, thylakoid, and stroma, and function in capturing light energy and fixing carbon dioxide [10]. Within chloroplast thylakoids, critical complexes such as photosystem II (PSII), cytochrome b6f (Cytb6f), photosystem I (PSI), and ATP synthase facilitate the conversion of solar energy into chemical energy [11,12]. In this intricate process, PSII and PSI perform oxygenic photosynthesis with electron shuttling facilitated by the cytochrome b6f (Cytb6f) complex [13,14]. Meanwhile, the ATP synthase complex utilizes the proton gradient formed via the electron transport chain to catalyze ATP synthesis [15]. Specifically, PSII is responsible for water molecule oxidation, generating electrons, while PSI accepts these electrons to reduce NADP+ [15]. PSII is a multisubunit complex encompassing D1 and D2 reaction centers alongside CP43 and CP47 light-harvesting proteins [15,16,17,18]. PSII is affected by a mutant of the multisubunit complex. For example, the N-terminus of D1 is a key oxidation site in the repair of PSII, which when mutated will lead to processive degradation [19]. The other protein, Psb28, interacts with the PSII core CP47 to form PSII core complexes [20]. PSI maintains remarkable structural conservation from cyanobacteria to eukaryotic plants and, with the assistance of the light-harvesting complex I (LHCI), forms a monomeric PSI-LHCI supercomplex in plants. This supercomplex is noted for its impressive energy transfer efficiency, which can famously approach 100% efficacy in facilitating charge separation [21,22,23]. In rice, LHCI-linked genes, such as *OsPGL1*, are critical for chlorophyll deficiency. *OsPGL1* is required for the effective assembly and targeting of the LHCIs in the PSI core complex of rice [24].

The thylakoid membrane’s intricate structure, distinctly separated into grana and stroma regions, is remarkable [25]. PSII is primarily located in the grana, while PSI is more abundant in the stroma lamellae [26]. Grana, comprising 10 to 20 thylakoid membrane layers, are crucial for optimizing photosynthetic efficiency owing to their extensive surface area and coalesced PSII units [27]. It is identified that grana’s architecture significantly contributes to the protection of PSII under excess light and assists in balancing excitation energy between PSII and PSI [28,29,30]. Grana formation is influenced by light intensity, thylakoid membrane protein complexes, and the chloroplasts’ ionic conditions [25]. In Arabidopsis, a novel ankyrin domain-containing protein, GDC1, is essential for forming grana [25]. In higher plants, ankyrin repeats are common proteins that interact between different groups of proteins, which is essential for a variety of biological functions and biochemical activities, including thylakoid development and stress responses. It is therefore important to identify new ANK genes and analyze their function [31].

In order to fill this gap in knowledge, our study focuses on a rice mutant with yellow leaves after treatment with ethyl methanesulfonate (EMS). We meticulously evaluated its phenotype, chloroplast anatomy, photosynthetic characteristics, ROS activity, and the expression of genes associated with senescence. Subsequently, our mapping and functional analysis designated the *YLD7* gene as essential for grana development in rice, which encodes an Ankyrin repeat-containing domain protein. 

## 2. Materials and Methods

### 2.1. Plant Materials and Growth Conditions

In this study, we induced the *yld7* mutation in *Oryza sativa* L. ‘Changchungu’ using ethyl methane sulfonate (EMS). ‘Changchungu’ was chose from the Chinese National Center for Rice Improvement and State Key Laboratory of Rice Biology, China National Rice Research Institute. Detailed gene mapping was facilitated through a cross with ‘Nipponbare’. The plants were cultivated under field conditions in Zhejiang and Hainan, China, spanning 2018 to 2023.

### 2.2. Paraffin Sectioning and Microscopic Analysis

Our protocol for paraffin sectioning was an adaptation of Zhao’s method [4]. We prepared tillering stage leaves by fixing, dehydrating, and embedding them in paraffin before cutting sections to a uniform thickness of 8 mm. The sections were stained with Safranin and Fast Green prior to examination under a Nikon SMZ1500 microscope (Nikon, Tokyo, Japan).

### 2.3. Measurement of Pigment Content and Photosynthetic Rate

Leaf samples were harvested from both wild-type and *yld7* plants, finely minced, and then extracted in 5 mL of 80% acetone in darkness for 48 h. Absorbance of the acetone-extracted pigments was read using a BECKMAN DU800 ultraviolet spectrophotometer [1,4]. The chlorophyll and carotenoid contents were quantified following Arnon’s [32] methods and using Wellburn and Alan (1994) calibrations [33]. In addition, photosynthetic rates for plants 65 days post-sowing were measured using a LICOR LI-6400 system (LICOR, Lincoln, NE, USA) under optimal conditions with 15 biological replicates.

### 2.4. Transmission Electron Microscopy (TEM)

We adhered to Chen et al.’s protocol [7], which includes steps for fixation, postfixation, dehydration, infiltration, and the staining of ultrathin sections, which is followed by observation using a Hitachi H-7650 microscope (Hitachi, Tokyo, Japan).

### 2.5. Histochemical Staining

Drawing from the protocols of Zhao and Chen [4,7], superoxide anion and peroxide were detected using nitro blue tetrazolium (NBT) and 3,3′-diaminobenzidine (DAB), respectively. Samples were subjected to bleaching post-staining for clarity. Trypan blue staining was applied to assess cell viability as outlined previously [34,35]. DAB and NBT staining were performed on leaves of wild-type and *yld7* plants to detect H_2_O_2_ and superoxide anion. The H_2_O_2_, ORF content, and CAT and POD activity of wild-type and *yld7* mutant leaves were measured using an assay kit (Suzhou Keming Biotechnology Co., Ltd. Suzhou, China). To detect nuclear DNA fragmentation, the terminal deoxynucleotidyl transferase dUTP nick-end labeling (TUNEL) assay was performed using the Fluorescein In Situ Cell Death Detection Kit (Roche, Basel, Switzerland) according to the manufacturer’s instructions. Methods for sectioning and fluorescence labeling were as described elsewhere [7,9].

### 2.6. Map-Based Cloning

Genetic analysis was conducted on F_1_ and F_2_ populations derived from reciprocal crosses between *yld7* and ‘Nipponbare’. The leaves of all the F_1_ plants resulting from the crosses were green and phenotypically identical to the wild type. Of the 1724 F_2_ plants that were derived from the F_1_ plants, 1298 showed the green leaf phenotype and 426 showed the yellow leaf phenotype. According to the *X*^2^ test, the wild-type to mutant phenotype segregation ratio was approximately 3:1 (*X*^2^ = 0.077, *P* = 78%).

Over 426 F_2_ mutant individuals with yellow leaves were selected for genetic mapping. Genomic DNA was extracted using the CTAB method [3]. The candidate genes were sequenced by Hangzhou Tsingke Biological Engineering Technology and Service Co., Ltd (Hangzhou, China).

### 2.7. Vector Construction

The construction of the CRISPR/Cas9 genome editing vectors was carried out as described by Wang [36]. For gene knockout, we designed the target sequences GCTGAACCAGGACATCCACC and CGGCGCCAAGTACGACGTCA. Primers for vector construction are listed in Additional File S3: Table S1. The fragment was inserted into a pC1300-UBI:Cas9 vector and introduced into ‘changchungu’ by Agrobacterium tumefaciens (EHA105)-mediated transformation. Three independent transgenic plants (T_0_) were obtained and sequenced. Three homozygous mutants and their wild type (‘changchungu’) were grown in the field for phenotype determination. For the subcellular localization of YLD7, we amplified the full-length *YLD7* coding sequence without the termination codon using primers YLD7-GFPF/R (Additional File S3: Table S1). The fragment was introduced into the green fluorescent protein (GFP) vector pCA1301-35S-S65T-GFP (CK-GFP), resulting in the pCA1301-35S-S65T-GFP-YPD1 (YPD1-GFP) construct. YLD7-GFP and the control vector CK-GFP were introduced into NPB rice protoplasts for the subcellular localization assay. The rice protoplasts were then observed using a Zeiss LSM700 laser scanning confocal microscope (Carl Zeiss, Inc., Thornwood, NY, USA).

### 2.8. Phylogenetic Analysis

Using BLASTP against YLD7, protein sequences were amassed for comparison. Alignment was performed with DNAMAN, and a neighbor-joining tree was created using MEGA v7, providing evolutionary insights through bootstrap analysis with 1000 replicates.

### 2.9. Quantitative Real-Time PCR

Total RNA was extracted from whole plants using TRIzol reagent (Invitrogen, Carlsbad, CA, USA). The cDNA was synthesized using 1 μg RNA and ReverTra Ace qPCR RT Master Mix with gDNA Remover (TOYOBO, Osaka, Japan) according to the manufacturer’s instructions. The qRT-PCR reactions were performed using a SYBR Green Kit (Applied Biosystems, Carlsbad, CA, USA). Ubiquitin was used as an internal control. Data were expressed as mean ± SD of three biological replicates. Student’s *t*-test was used for statistical analysis.

### 2.10. Immunoblot Analysis

First, we performed total protein extraction from wild-type and *yld7* at the seedling stage. Tissues were ground in liquid nitrogen and thawed in extraction buffer [50 mM Tris-HCl pH 7.5, 150 mM NaCl, 10% glycerol (*v*/*v*), 0.1% Nonidet P-40, 1 mM DTT, 1 mM PMSF and 1× complete protease inhibitor cocktail (Roche)] for 15 min on ice. The supernatant was collected by centrifugation at 12,000× *g* for 10 min at 4 °C. Total proteins were separated by SDS-PAGE gels (8%), transferred to polyvinylidene difluoride (PVDF) membranes (GE Healthcare, New York, NY, USA), and blotted with different primary antibodies. Antibodies against photosystem proteins (anti-Lhca1, anti-Lhca2, anti-Lhca3, and anti-Lhca4) were obtained from Agrisera (Beijing, China) [37]. Anti-β-actin was used as control.

### 2.11. RNA-Seq Analysis

Total RNA was extracted from wild-type and *yld7* at the seedling stage. mRNA was purified from total RNA using poly-T oligo from total RNA using poly-T oligo-attached magnetic beads. cDNA was synthesized using random hexamer primers. The library was constructed and sequenced using an Illumina Hisequation 2000 (Novogene, Tianjin, China). The significance of differentially expressed genes (DEGs) was determined using log2 (fold change) > 1 and q values < 0.05. Gene ontology analysis was performed using GOseq. Pathway enrichment analysis was performed using the Kyoto Encyclopedia of Genes and Genomes database.

### 2.12. Statistical Analysis

All data were expressed as mean ± SD from at least three biological replicates. Statistical significance was determined using Student’s *t*-test with *p*-values < 0.05 considered significant.

## 3. Results

### 3.1. Phenotypic Analysis Reveals Yellowing and Dwarfism in yld7 Mutants

**Figure 1:**
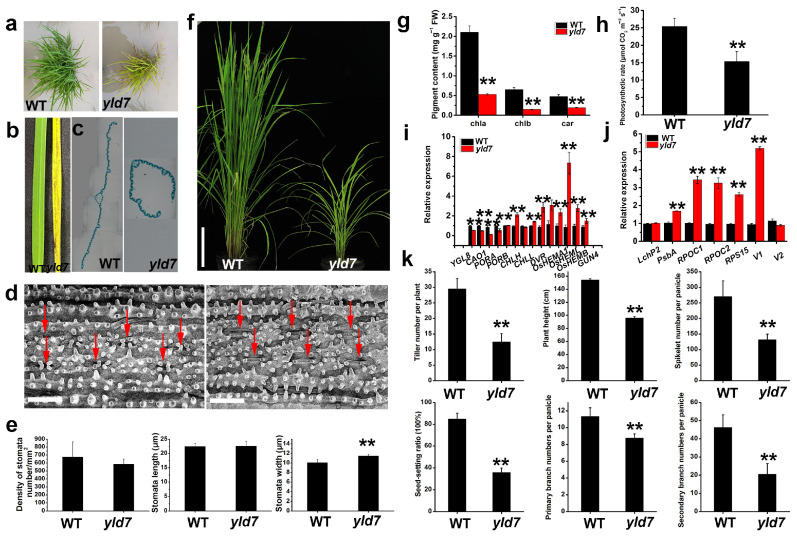
Phenotypic comparison of wild-type (WT) and *yld7* plants. (**a**) Morphological differences in WT and *yld7* at seedling stages. (**b**), Leaf phenotype of WT and *yld7* plants at seedling stages (the youngest fully expanded leaf). (**c**) Cross-sections of leaf of WT and *yld7* at seedling stage. (**d**) Stomatal density comparison with red arrows marking stomata. Bars = 100 μm. (**e**) Statistical data on stomatal length (**d**) and width (**e**), with three and twenty independent replicates, respectively (** *p* < 0.01). (**f**) Morphological differences in WT and *yld7* at tillering stages. (**g**) Chlorophyll content analysis. Error bars represent SD, n = 10 (** *p* < 0.01). (**h**) Comparison of photosynthetic rates. Error bars denote SD, n = 15 (** *p* < 0.01). (**i**,**j**) Gene expression related to chlorophyll synthesis (**i**) and chloroplast development (**j**) at the tillering stage. (**k**) Statistical data on tillering number, plant height, grains per panicle, setting rate, primary branch, and secondary branch in both plant types. Values are means ± SD of three biological replicates (** *p* < 0.01, Student’s *t*-test).

The mutants displaying yellowing and a lesion-like phenotype from the seedling stage through to maturity were derived from EMS-treated indica rice CV. Changchungu (Figure 1a,b,f). These mutants also exhibited reduced leaf size and vein width (Figure 1c) along with a 14% increase in stomatal width as compared to the wild-type (WT) plants (Figure 1d,e). However, there were no significant changes in stomatal density or length (Figure 1d,e). The *yld7* mutants had considerably lower levels of chlorophyll a, chlorophyll b, and carotenoids, which led to a decreased photosynthetic rate (Figure 1g,h). Leaf senescence is characterized by changes in gene expression related to chlorophyll synthesis and chloroplast development [4]. A comparative gene expression analysis revealed notably lower expression levels of key chlorophyll synthesis and chloroplast development genes in *yld7* plants, suggesting impaired chloroplast formation in the mutants (Figure 1i,j). Compared to WT, *yld7* mutants displayed a significant reduction in growth parameters: 42% fewer tillers, 62% shorter stature, 49% fewer grains per panicle, a 42% lower setting rate, and reduced primary (77%) and secondary (44%) branch numbers (Figure 1k). These findings demonstrated that mutations in the *YLD7* gene cause leaf senescence and affect overall plant growth.

### 3.2. Premature Cell Death in yld7 Plants

**Figure 2:**
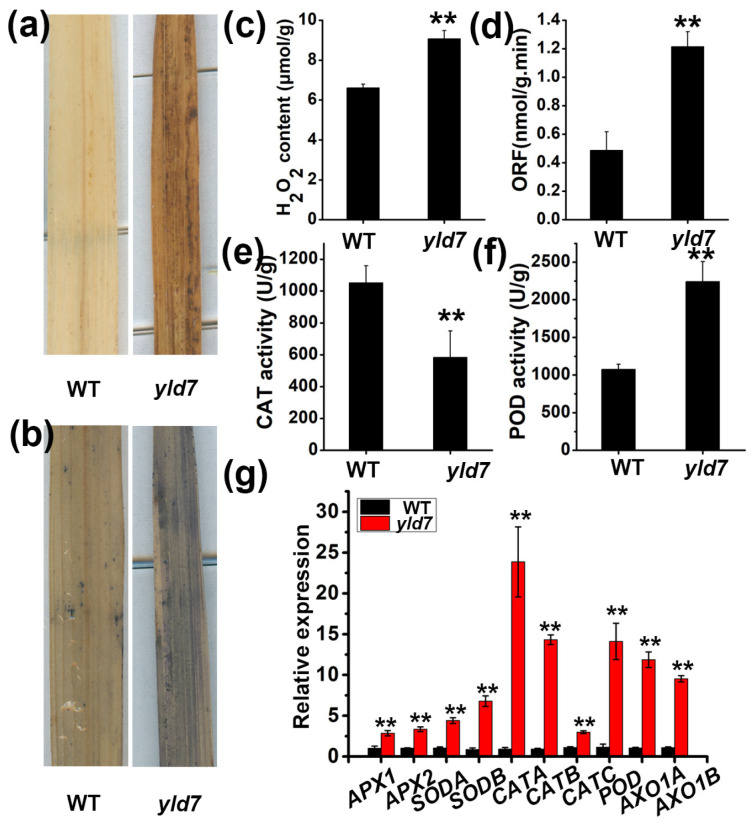
ROS accumulation in WT and *yld7* plants. (**a**,**b**) DAB and NBT staining at the seedling stage. (**c**–**f**) Quantitative analysis of H_2_O_2_, ORF content, and CAT, POD enzyme activities. Mean ± SD from five replicates (** *p* < 0.01). (**g**) Relative expression of ROS detoxification genes. Error bars represent SD, n = 3 (** *p* < 0.01, Student’s *t*-test).

Reactive oxygen species (ROS) are known to trigger premature cell death and leaf senescence [7,38]. We therefore identified ROS in the upper leaves (seedling stage) of both WT and mutant plants using NBT and DAB staining techniques. In *yld7* mutants, extensive DAB and NBT staining indicated increased ROS levels (Figure 2a,b). We measured the concentrations of senescence-related substances, such as the derived ROS. We observed higher concentrations of hydrogen peroxide (H_2_O_2_) (9.08 μmol/g and 6.61 μmol/g, respectively) and oxygen free radicals (ORFs) (1.21 and 0.49 nmol/g.min, respectively) in *yld7* leaves than in wild-type leaves (Figure 2c,d), showing the accumulation of ROS in *yld7* mutants. Catalase (CAT) and peroxidase (POD) are important ROS scavenging enzymes that have crucial regulatory functions in plant senescence [39]. The activity of CAT was significantly lower in *yld7* (1051 U/g fresh weight and 585 U/g fresh weight, respectively) compared to the wild type (*p* < 0.05, Student’s *t*-test; Figure 2f,g). Conversely, POD activity was significantly higher in *yld7* leaves compared to wild-type leaves (2242 U/g fresh weight and 1077 U/g fresh weight, respectively) (Figure 2e,f). Since ROS scavenging systems play an important role in ROS detoxification [40], we identified the expression levels of genes involved in ROS scavenging. The levels of ROS scavenging genes were significantly higher in *yld7* plants compared to the wild type (*p* < 0.01, Student’s *t*-test; Figure 2g).

### 3.3. TUNEL Assay for Cell Death in yld7 Plants

**Figure 3:**
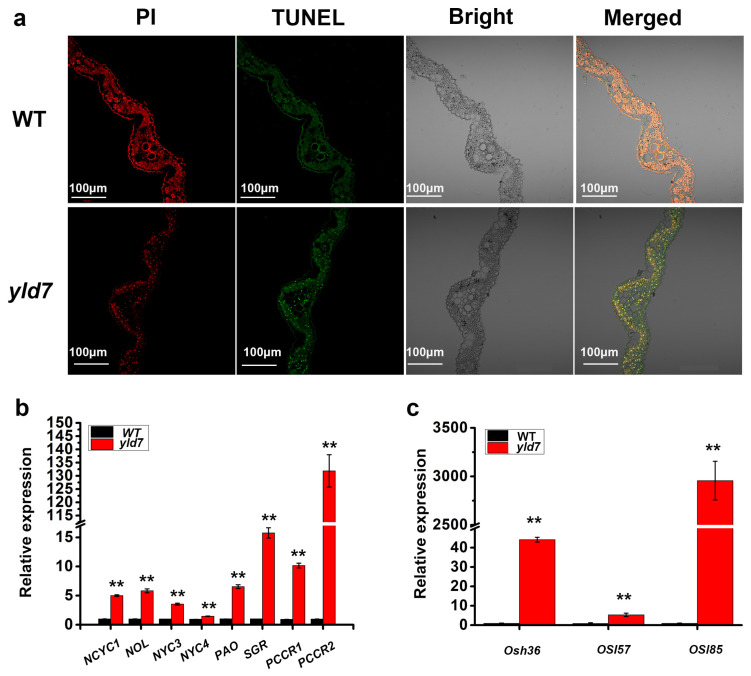
Cell death detection in WT and *yld7* plants. (**a**) Cell death detection in *yld7* plants. Bars = 100 μm (**b**,**c**) Expression analysis of CDGs (**b**) and other SAGs (**c**). Error bars represent SD, n = 3 (** *p* < 0.01, Student’s *t*-test).

We noted that the mutants exhibited an early onset of lesion-like leaf in the paddy field, leading us to speculate that the *yld7* mutation caused cell death in rice. We conducted a TUNEL assay to investigate this further by examining cell death in *yld7* plants first. When a cell undergoes death, the TUNEL reagent can be used to attach fluorescein to the exposed 3′-OH of the broken DNA strands, which can then be detected through fluorescence microscopy [7,9]. A TUNEL assay revealed stronger signals in *yld7* lesion-like leaves, indicating higher rates of cell death compared to WT (Figure 3a). RT-qPCR analysis showed an elevated expression of chlorophyll degradation genes (*NCYC1*, *NOL*, *NYC3*, *NYC4*, *PAO*, *SGR*, *RCCR1*, *RCCR2*) and other senescence-associated genes (*Osh36*, *Osl57*, *Osl85*) in *yld7* plants (Figure 3b,c). These results indicate enhanced leaf senescence in *yld7* mutants compared to WT.

### 3.4. Map-Based Cloning and Candidate Analysis of the YLD7 Gene

**Figure 4:**
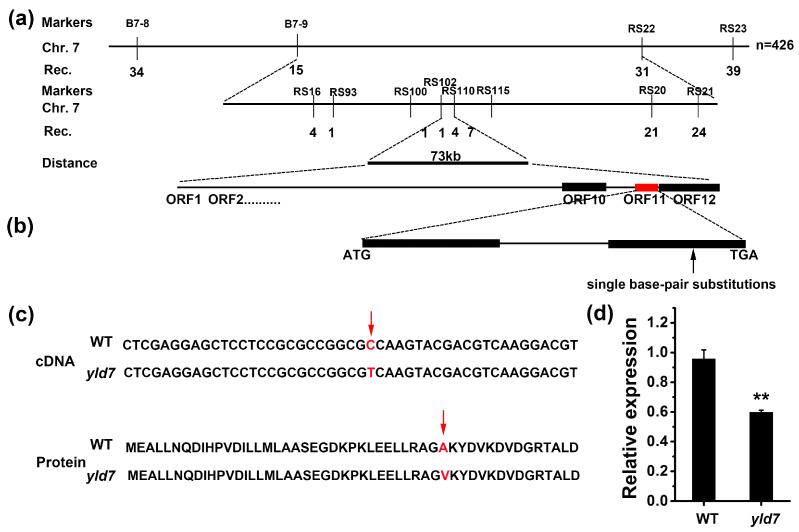
*YLD7* candidate gene analysis. (**a**) *YLD7*’s chromosomal location with marker data. Numbers indicate recombinants. (**b**) Structure and sequence variations in LOC_Os07g33660 between WT and *yld7*. (**c**) Genome and protein-level mutations with red arrows marking the site. (**d**) *YLD7* transcript levels at the 3-leaf stage, normalized to rice ubiquitin. Error bars represent SD, n = 3 (** *p* < 0.01, Student’s *t*-test).

**Figure 5:**
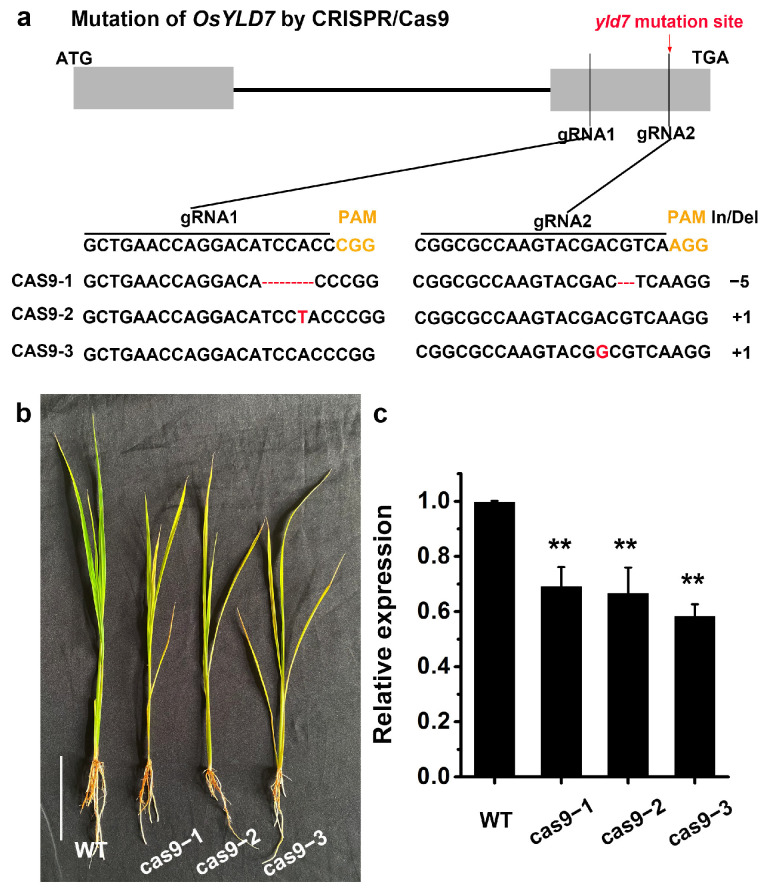
*YLD7* targeted deletion via CRISPR/Cas9. (**a**) Confirmation of *YLD7* deletion in WT with Cas9/sgRNA constructs. Target sequence highlighted, PAM site in yellow. Altered sequences in red. (**b**) Phenotypes of mutants with *YLD7* Cas9/sgRNA constructs (bar = 1 cm). (**c**) *YLD7* transcript levels in WT and transgenic plants. Error bars represent SD, n = 3 (** *p* < 0.01, Student’s *t*-test).

To elucidate the genetic underpinnings of the *yld7* phenotype, a map-based cloning approach was undertaken. In an F_2_ population derived from a cross between the *yld7* mutant and the japonica variety NPB, segregation analysis implied that a single recessive nuclear gene was responsible (Figure 4a). Further high-resolution mapping and DNA sequencing pinpointed a critical single nucleotide substitution (C to T) in the LOC_Os07g33660 gene, causing a key amino acid substitution (Figure 4b,c). This gene’s transcript levels were significantly diminished in *yld7* mutants (Figure 4d). CRISPR/Cas9-mediated targeted deletion of LOC_Os07g33660 in WT rice produced mutants with a similar phenotypic presentation, validating this gene’s involvement in the *yld7* phenotype (Figure 5a–c).

### 3.5. YLD7 Encodes an Ank-Repeat Containing Protein

**Figure 6:**
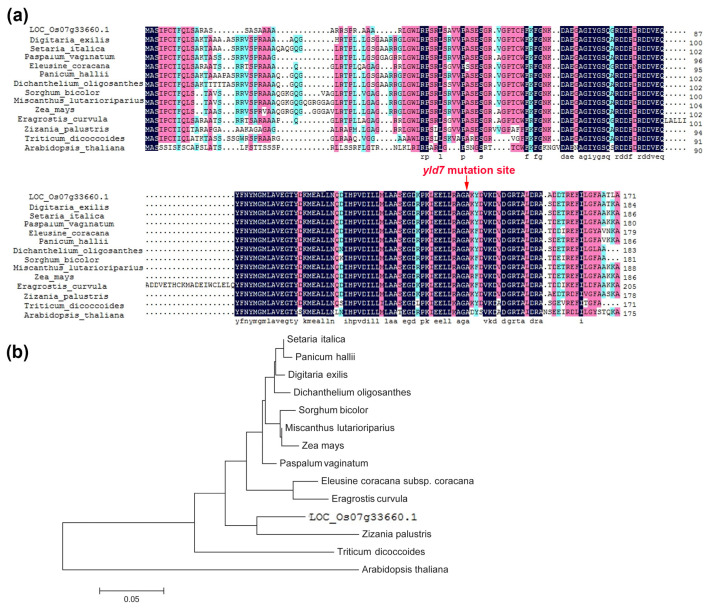
Phylogenetic analysis of YLD7 homologues. (**a**) Amino acid sequence alignment with conserved residues shaded. Amino acids that were fully or partially conserved are dark blue and pink, respectively. (**b**) Phylogenetic tree constructed using MEGA 7.0, showing evolutionary relationships. The scale bar represents the percentage of substitutions per site.

The *YLD7* gene, consisting of two exons, encodes a protein with Ank repeats. BLASTP analysis showed YLD7’s involvement in LHCP translocation defects and revealed its conservation across various plant species including Digitaria exilis and Arabidopsis thaliana (Figure 6a). Phylogenetic analysis classified YLD7 homologs into monocots and dicots with YLD7 falling in the monocot group, underscoring its evolutionary significance (Figure 6b). These findings illustrate that YLD7 is highly conserved in plants, thus implying its fundamental role in plant growth and development.

### 3.6. Subcellular Localization of YLD7 Protein

**Figure 7:**
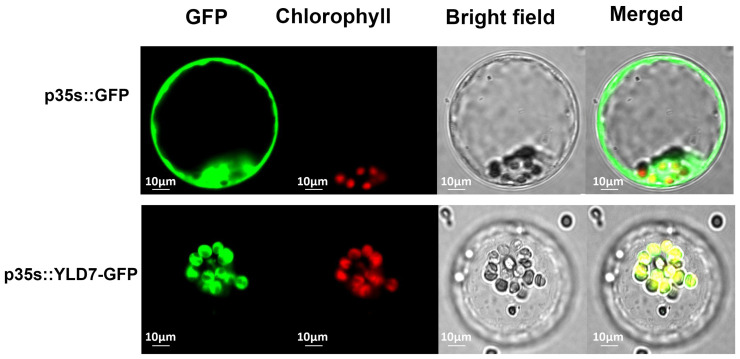
Subcellular localization of YLD7-GFP. i. Control GFP image. ii. YLD7-GFP fusion localized to the chloroplast (bar = 10 μm).

To investigate the subcellular localization of YLD7, rice protoplasts were transformed with the YLD7-GFP plasmid. As a control, the CK-GFP vector was also used (Figure 7). The green fluorescent signal of the YLD7-GFP construct coincided with the autofluorescence of the chloroplast. Based on these observations, it can be inferred that YLD7 localizes to the chloroplast.

### 3.7. YLD7 Protein Is Essential for the Formation of Grana and Stroma

**Figure 8:**
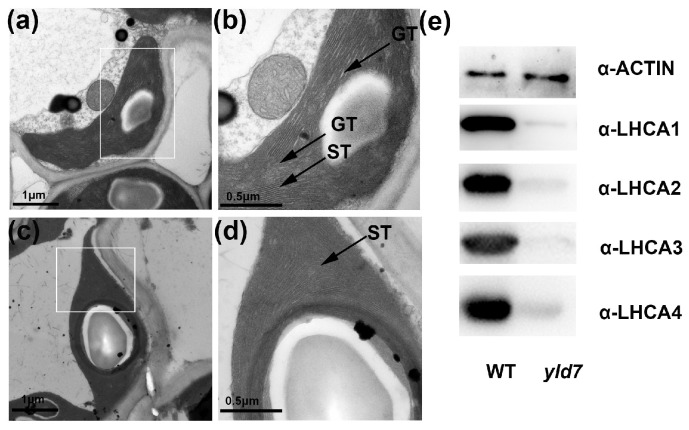
Chloroplast ultrastructure in WT and *yld7*. (**a**) WT chloroplasts. Bars = 1 μm. (**b**) Close-up of WT chloroplast, highlighting stromal thylakoids (ST). Bars = 0.5 μm. (**c**,**d**) Abnormal chloroplasts in *yld7*. Bars = 1 μm and 0.5 μm, respectively. GT, Grana thylakoid stacks; ST, stromal thylakoids. (**e**) Immunodetection of thylakoid proteins with specific antibodies.

Previous reports have shown that GDC1 is linked to stacking stromal thylakoids together to create appressed grana [25]. Transmission electron microscopy (TEM) revealed that compared to the well-structured chloroplasts of WT, the *yld7* mutants’ chloroplasts were aberrantly formed with many stromal thylakoids failing to compose properly appressed grana (Figure 8a–d). In addition, immunoblot analysis of the four LHCI proteins (LHCA1, LHCA2, LHCA3 and LHCA4) showed a significant reduction in these proteins in *yld7* (Figure 8e). Collectively, these observations underscore the vital role of YLD7 in the assembly of both grana and stroma.

### 3.8. RNA-Seq Analysis Reveals Altered Gene Expression in yld7 Mutants

**Figure 9:**
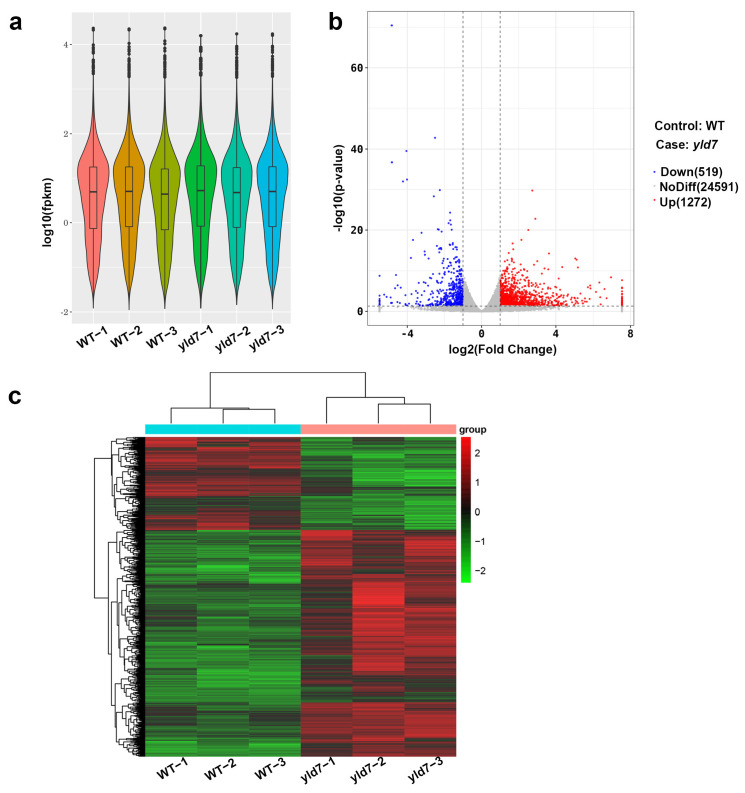
RNA-seq analysis in WT and *yld7*. (**a**) Transcript expression comparison. (**b**) Volcano plot of gene expression changes. Red represents highly expressed genes. Blue represents low expressed genes. (**c**) Cluster analysis of differentially expressed genes. Red represents highly expressed genes. Green represents low expressed genes.

RNA sequencing was performed to assess the effect of the *yld7* mutation on gene expression using yellow leaves from *yld7* and green leaves from wild-type (WT) plants at the seedling stage. With three replicates, high consistency among biological replicates was evident from the transcript level box plots (Figure 9a). A total of 26,382 genes were analyzed, revealing 1272 up-regulated and 519 down-regulated genes in *yld7* compared to WT (Figure 9b,c, Additional Files S2). Analyses of the Gene Ontology and the Kyoto Encyclopedia of Genes and Genomes suggested changes in pathways related to oxidation–reduction, oxidoreductase activity, and starch and sucrose metabolism in *yld7* (Figures S1–S3).

### 3.9. Haplotype Analysis of YLD7

**Figure 10:**
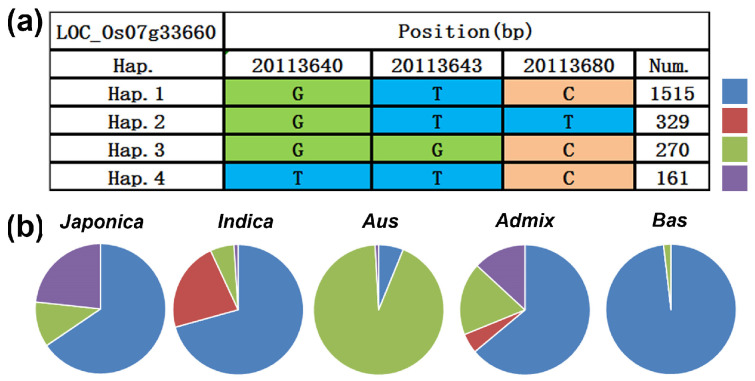
*YLD7* genetic diversity analysis. (**a**,**b**) SNP variation types in *YLD7* CDs across 3127 accessions. Top panel shows the SNP variants (**a**), and the bottom panel displays the distribution of these variants across different rice subspecies (**b**).

Investigating SNP data from the 3K rice genome project, we found four SNP variants (GTC, GTT, GGC, TTC) within the first exon of the *YLD7* gene (Figure 6a and Figure 10a). Distribution patterns showed subspecies-specific variation: japonica, admix, and Bas predominantly featured the GTC variant, while indica and Aus subspecies mostly carried GTT and GGC variants, respectively (Figure 10b).

## 4. Discussion

This study is the initial report of isolating the *YLD7* gene in rice via the *yld7* mutant and has established that the *YLD7* coding sequence encodes an Ank-repeat protein, which is essential in grana formation. Our TEM analysis underpins the critical role of YLD7 in this process, while subcellular localization studies connect *YLD7* to chloroplast biogenesis. The reduced levels of Lhca1-4 proteins in *yld7* mutants further implicate *YLD7* in the import of LHCP into the thylakoid membrane of chloroplasts.

### 4.1. YLD7 Act as a Bridge in the Plant Thylakoid Membrane for Grana and Stroma Formation

The gene *YLD7* serves as an integral component in the thylakoid membrane, supporting the organization of grana and stroma. An increasing amount of genes associated with abnormal chloroplasts have been cloned and characterized for function in rice [4,7,9,25,41]. The thylakoid membrane located in the chloroplasts is crucial for the metabolism of plant pigments. Disruptions to its internal structure can lead to abnormal coloration in the leaves [42]. Chlorophyll-deficient mutants can be classified into two categories based on their chlorophyll content and chlorophyll a/b ratio. The first category, referred to as grana-rich mutants, have chlorophyll a/b ratios lower than the wild type, and most of their lamellae are stacked into grana. The second category, known as grana-deficient mutants, exhibit high ratios of chlorophyll a/b and low chlorophyll content with very few grana in their chloroplasts [25,43]. The chloroplast-localized SDR protein NYC1, a short-chain dehydrogenase/reductase, has a pivotal function in the regulation of LHCII and the degradation of thylakoid membranes during senescence [44]. *Oschlh* mutants result in an underdeveloped thylakoid membrane in the chloroplasts of the mutants [45]. The Chl deficit observed in the *ygl1* mutant may be attributed to the delayed formation of thylakoid membranes, and the underdeveloped chloroplast led to a reduction in Chl accumulation in the ygl1 seedling stage [46]. The *pgl* leads to a lower chlorophyll content, which is coupled with a disordered thylakoid ultrastructure that diminishes photosynthesis. This results in lower grain yield and quality [47]. An insertion mutation of *YELLOW-GREEN LEAF2*, which encodes *Heme Oxygenase 1*, leads to a deficient stacking of grana [48]. The mutant plants, papst1, exhibited a thylakoid development deficiency, which caused leaf chlorosis during the early leaf developmental phase. However, leaf growth returned to normal 4 to 6 days after leaf emergence [49]. In Arabidopsis, a novel ankyrin domain-containing protein, GDC1, which is essential for the formation of grana, was identified [25]. Phylogenetic analysis revealed a high conservation of YLD7 with GDC1, indicating that *YLD7* may also be essential for Grana formation. Haplotype analysis of *YLD7* indicates that the Ank repeat is extremely conserved and haplotype SNPs are only found in non-Ank repeat regions (Figure 6a and Figure 10a), whereas the mutant *yld7* mutation site in this study is only in the Ank repeat region (Figure 4a,b).

### 4.2. yld7 Accelerated Senescence and Cell Death in the yld7 Mutants

A decrease in chlorophyll levels and an increase in several types of ROS are typically associated with leaf senescence. [4,7]. Our investigation showed a decline in the chlorophyll content and photosynthesis rate in the *yld7* mutant’s leaves (Figure 1g,h). Furthermore, the expression levels of genes involved in chlorophyll biosynthesis and chloroplast development were notably reduced in the *yld7* plants compared to those in the wild type (Figure 1i,j). Therefore, it is postulated that the degradation of chlorophyll and chloroplasts plays a significant part in leaf senescence in *yld7* plants. Numerous metabolic procedures are involved. Reactive oxygen species (ROS) can lead to oxidative harm to thylakoid membranes and other cellular components [4,50]. Compared to wild-type leaves, *yld7* leaves exhibited a substantial increase in OFRs, a type of ROS (Figure 2), and recorded higher levels of POD activity than wild-type plants (Figure 2). These results make it evident that leaf senescence in *yld7* plants is linked to ROS. Trypan blue and TUNEL assays indicated a patchy arrangement of cell death in leaf mesophyll cells of the *yld7* mutants (Figure 3a), and more robust TUNEL signals were visible in the *yld7* leaves (Figure 3b); however, hardly any signals were identified in the leaves of the WT. These outcomes imply that *yld7* could participate in a chloroplast-induced senescence of leaf in rice.

We used the map-based cloning technique to clone *yld7* and validated its function in transgenic plants. The *YLD7* gene comprises two exons and encodes a protein containing Ank-repeat. Ankyrin repeats represent one of the most prevalent protein sequence motifs, which is found in prokaryotes, eukaryotes and some viruses [51]. Most ankyrin proteins possess over two ankyrin repeats and are believed to facilitate interactions between different groups of proteins [52,53], Currently, there is limited research on the function of proteins containing a single ankyrin domain. *YLD7* is an example of such a protein, which is located in the chloroplast (Figure 7). The homologous protein GDC1 has also been found in the chloroplast of Arabidopsis thaliana [25]. TEM results indicated that the grana formation was lost in *yld7*. The abnormality of the thylakoid in *yld7* leads to a reduction in the photosynthesis rate. Plastid-encoded proteins of LHCA1, LHCA2, LHCA3, and LHCA4 were decreased in *yld7*. Two pathways, PSI and PSII, are responsible for electron transfer during photosynthesis. Lhca1–Lhca4 form a PSI-LHCI super complex for the collection of light energy [15]. The protein levels of LHCA1, LHCA2, LHCA3, and LHCA4 in *yld7* may be inhibited by PSI, indicating that photosynthesis was influenced in *yld7* plants. The reduced plant height was linked to the decreased photosynthesis in *yld7* plants. This discovery emphasizes YLD7′s vital roles in the thylakoid arrangement, grana structure, and the senescence of leaves. A yellow leaf mutation in rice, *ygl*, was shown to reduce chlorophyll content but increase PSII efficiency. Thus, mutants with yellow leaves but high photosynthesis could be used for rice breeding [54]. The identification of *YLD7* as a key regulator of chloroplast development and leaf senescence offers valuable insights for rice breeding programs. By manipulating the *YLD7* gene or its homologs, it may be possible to develop rice varieties with enhanced photosynthetic efficiency and delayed senescence, improving grain yield and crop resilience. Future studies should focus on identifying the proteins that interact with *YLD7* to further elucidate its role in thylakoid membrane organization and ROS regulation.

## 5. Conclusions

**Figure 11:**
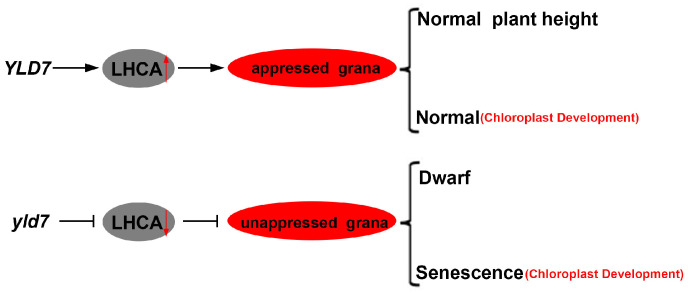
Simplified schematic model in YLD7. Arrow: activate; Bar: repress. Down red arrow: down-regulation; Up red arrow: up-regulation.

In conclusion, *YLD7* is a critical regulator of chloroplast development and leaf senescence in rice. We propose a putative model based on our findings (Figure 11). Our study demonstrates that *YLD7*, an ankyrin domain-containing protein, is essential for grana formation and plays a protective role in mitigating oxidative stress during leaf aging. The *yld7* mutant provides a valuable model for studying the molecular mechanisms underlying chloroplast biogenesis and senescence. These findings have important implications for rice breeding strategies aimed at improving photosynthetic efficiency and crop yield. Further research is needed to fully understand the regulatory networks in which *YLD7* participates and to explore its potential for improving agronomic traits in rice.

## Data Availability

Data will be made available on request.

## Supplementary Materials

The following supporting information can be downloaded at https://www.mdpi.com/article/10.3390/genes15101267/s1, Additional File S1: Gene Ontology and Kyoto Encyclopedia of Genes and Genomes (KEGG) pathway analyses. Figure S1. Statistics of GO enrichment analysis of differential expression genes between wild-type(WT) and *yld7* plants. mRNA was purified from total RNA isolated from tillering-stage plants of WT (‘changchungu’) and *yld7*. Figure S2. Statistics of pathway enrichment analysis of differential expression genes between wild-type (WT) and *yld7* plants. mRNA was purified from total RNA isolated from tillering-stage plants of WT (‘changchungu’) and *yld7*. Figure S3. The number of differential expression genes involved in different biological processes, cellular, components and molecular functions. mRNA was purified from total RNA isolated from tillering-stage plants of WT (‘changchungu’) and *yld7*; Additional File S2: Total DEGs were identified in *yld7* compared to WT; Additional File S3: Table S1. Primers used in this paper.
