# Supplementary material for: YELLOW LEAF AND DWARF 7, Encoding a Novel Ankyrin Domain-Containing Protein, Affects Chloroplast Development in Rice
Source: Genes (Basel). 2024 Sep 27;15(10):1267. doi: 10.3390/genes15101267 (PMC11507589; doi:10.3390/genes15101267)
Supplement: Supplementary file 1 [file genes-15-01267-s001.zip › Additional file 3.pdf]

Table S1. Primers used in this paper.

Primer Name	Primers Sequence (5'-3')
B7-8F	TGTGGACAACCTCAACTGAAAGC
B7-8R	CATAATCACCAACATCGGAGAAGC
B7-9F	TTCTGCTCCACGTGTTCTTG
B7-9R	TAACCCATAGTCCCGTACGC
RS16F	TGTTGGACGGAAGTAAAGCC
RS16R	AGGTATCTCACCATCGCTCA
RS20F	TAGCCTTCATTCCTAGCTAT
RS20R	GTCATATACGTACGTGCAAA
RS21F	ATTCCGCCCAGAGGAAGAGTACG
RS21F	CTGCATGCCACCACACAAACC
RS22F	GTTGCTACTGCCCTTATT
RS22R	AAGCCTTGTGAAGTGAGTA
RS23F	ACAGTATCCAAGGCCCTGG
RS23R	CACGTGAGACAAAGACGGAG
RS93F	AGGAGGCCGAAAAATCAGTT
RS93R	TAAGGGCAAATGTGCAACAA
RS100F	CGAGCTATCGATGTGTTTCG
RS100R	TTGGCCCATTGTCAGTTGTA
RS102F	CCTATCTCGTGTGGCTCCAT
RS102R	CCGATCGTTTCGCTTATTCT
RS110F	TCAATGGTTTGCTTTGCTTG
RS110R	AAATGGAAGCCAGTTCAACG
RS115F	GCAGAAGAGGAAACCGTGAG
RS115R	CCTGGAGGCCTGGACATATAC
qNYC1F	CATGCAACACCAACAAAAGG
qNYC1R	GACCATTCCAGGAGAAGCAG
qNOLF	CCACGAAAGGTATAGGATATG
qNOLR	TCAAGTCAGTCACCGCAGAT
qNYC3F	TCTATCTAGGTGCCAAAGGC
qNYC3R	ATTCTGGCACCTGCTGTTTC
qNYC4 F	CGTCTATGACCAACTCATGG
qNYC4R	TGCGTCAGCTCTGTATTGCT
qPAOF	AAGCCTCCGATGTTACCGAA
qPAOR	CGAGGGTTTCCAGAATTTGA
qSGRF	GCAATGTCGCCAAATGACG
qSGRR	GCTCACCACACTCATTCCCTAAAG
qRCCR1F	GGATCGACGATTGATTTCATG
qRCCR1R	GTCGAGGCGTTCAGAAAGAT
qRCCR2F	TGGCGAGGGACAGGAAGGT
qRCCR2R	GGATGTGGTGGCGAGAGAAAC
qLch P2F	GAAGAAGATCAAGAACGGCC

qLchP2R	TTGCCGGGGACGAAGTTGGT
qPsbAF	AGAGACGCGAAAGTACAAGC
qPsbAR	AAGTTGCGGTCAATAAGGTA
qRpoC1F	TCCGTCGGAACAACAATCTTG
qRpoC1R	TCCACGGCTTCTTGTACCAAT
qRpoC2F	ATGCATCGCAGGTACACCAA
qRpoC2R	CCCTCGCGTAAATTGCTTTG
qRps15F	AGATACGGAGACTTGCTTCA
qRps15R	GCTCCCTAATATCCAACTGACT
qV1F	AGAATCAGCGCGAGAAGAGAACCT
qV1R	TACACCAGCTTTGGAGGAGCTGAA
qV2F	AGCAGATCCGTGATTACATGGCGA
qV2R	TGCCTCTTCACTCTCTGCAACCAA
qAPX1F	AGGTGCCACAAGGAAAGATCTGT
qAPX1R	TCAGCAGGGCTTTGTCACTAGGAA
qAPX2F	TGGGAAGATGCCACAAGGAGAGT
qAPX2R	TCCGCAGCATATTTCTCCACCAGT
qSODA1F	ATCTGGATGGGTGTGGCTAGCTTT
qSODA1R	AGTACGCATGCTCCCAGACATCAA
qSODBF	TCCGCCGTATAAACTTGATGCCCT
qSODBR	TGGGTTGCCGTTGTTGTATGCTTC
qcatAF	CAACCGCAACGTCGACAACTTCTT
qcatAB	TTCACCGGCAGCATCAGGTAGTTT
qcatBF	GCTTGCTTTCTGCCCAGCGATAAT
qcatBR	AAATAGTTTGGGCCAAGACGGTGC
qcatCF	AGAAGGTGGTGATTGCCAAGGAG
qcatCR	TCTCTTGATGAACCGGTCTTGCCT
qPOD1F	ACGTCGGGGTCGCCAACAAC
qPOD1R	CGAACTCGTCCACCGACGCC
qAOX1aF	CTTCGCATCGGACATCCATTA
qAOX1aR	TCCTCGGCAGTAGACAAACATC
qAOX1bF	CCTGCTCAGTTCATCACCATCA
qAOX1bR	GCATAAAACGGAGTGACAATAGC
qYGL8F	TGGATCTAACATGACACGCACCCA
qYGL8R	ACTGTAACGGCATTCTTCTCCGGT
q CAO1F	TTGGCTCAGTTAATGAGGGCAGAATCC
q CAO1R	GGATGCGCACGTTGAGCATCTTTGTGG
qPORAF	ATGGCTCTCCAAGTTCAG
qPORAR	TGGCTCACGCTAAGGAAC
qPORBF	CCGCAAGGAGGGAGCGGTG
qPORBR	CCCTCTTGGTGCTAAGGCCG
qCHLHF	GCACGGGAACTTGGCGTTTCATTA
qCHLHR	ACATGTCCTGGAGCTGCTTCTCAT
qCHLDF	TAGCACAGCTGTCAGAGTGGGTTT

qCHLDR	TTGCCAGCCACCTCAAGTATCTCA
qCHLIF	AGGGATGCTGAACTCAGGGTGAAA
qCHLIR	AAGTAGGACTCACGGAACGCCTTT
qDVRF	AGCCCAGGTTCATCAAGGT
qDVRR	TGATCACCCTCTCGAAGAACT
qOsCHLMF	GCTTCATCTCCACGCAGTTCTACT
qOsCHLMR	GCAATGACGAATCGAAGACGCACA
qYGL1F	CCAGCCACTGATGAAAGCAGCAAT
qYGL1R	AGAGCGCTAATACACTCGCGAACA
qOsHEMA1F	GATGCAATCACTGCTGGAAAGCGT
qOsHEMA1R	CCATCTTGCCAGCACCAATCAACA
qOsHEMLF	AGAACAAAGGGCAGATTGCTGCTG
qOsHEMLR	TGTTTCGTCAAGTCACGGAGAGCA
qOsHEMBF	TGGCATTGTCAGGGAAGATGGAGT
qOsHEMBR	CCAAAGCAGCACGTATTGCTCCAA
qGUN4F	AAGGGAAGGAGAGGCCAAAGTTCA
qGUN4R	ACCATGACCAGCATCTCTGCATCA
qOsh36F	CCTGGTGATCTGAAGGTTGT
qOsh36R	CATGGCAACCAGTGTAAAGC
qOsl57F	ACCCTAAAGTAAATGAAGTC
qOsl57R	CCTGCTCTTGTCTTGTTA
qOsl85F	GAGCAACGGCGTGGAGA
qOsl85R	GCGGCGGTAGAGGAGATG
YLD7-SalI-rGFP-FP	caattactatttacaattacagtcgacATGGCATCCATCCCGTGCACC
YLD7-SalI-rGFP-RP	cccttgctcaccatggatcctctagaGGCGGCCAAGGTGGCGGCGAAG
YLD7gRNA1	GCTGAACCAGGACATCCACC
YLD7gRNA2	CGGCGCCAAGTACGACGTCA
qYLD7F	GACGTCGAGCAGTACTTCAACT
qYLD7R	GCGCCTCCATCTTGTCGTA
UbiquitinF	AACCAGCTGAGGCCCAAGA
UbiquitinR	ACGATTGATTTAACCAGTCCATGA
