# Supplementary material for: YELLOW LEAF AND DWARF 7, Encoding a Novel Ankyrin Domain-Containing Protein, Affects Chloroplast Development in Rice
Source: Genes (Basel). 2024 Sep 27;15(10):1267. doi: 10.3390/genes15101267 (PMC11507589; doi:10.3390/genes15101267)
Supplement: Supplementary file 1 [file genes-15-01267-s001.zip › Additional file 1.pdf]

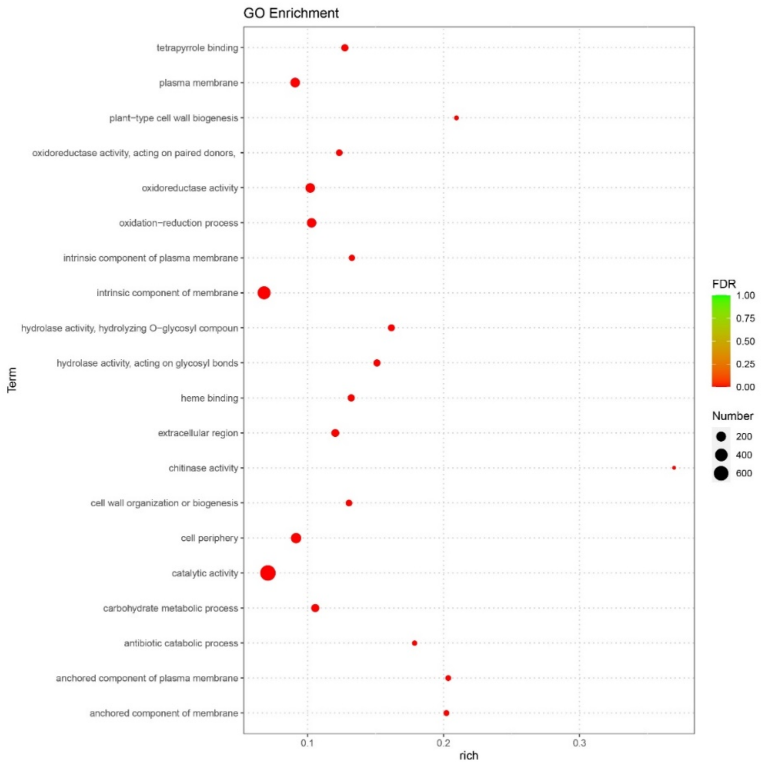

Figure S1. Statistics of GO enrichment analysis of differential expression genes between wild-type(WT) and *yld7* plants. mRNA was purified from total RNA isolated from tillering-stage plants of WT ('changchungu') and *yld7*.

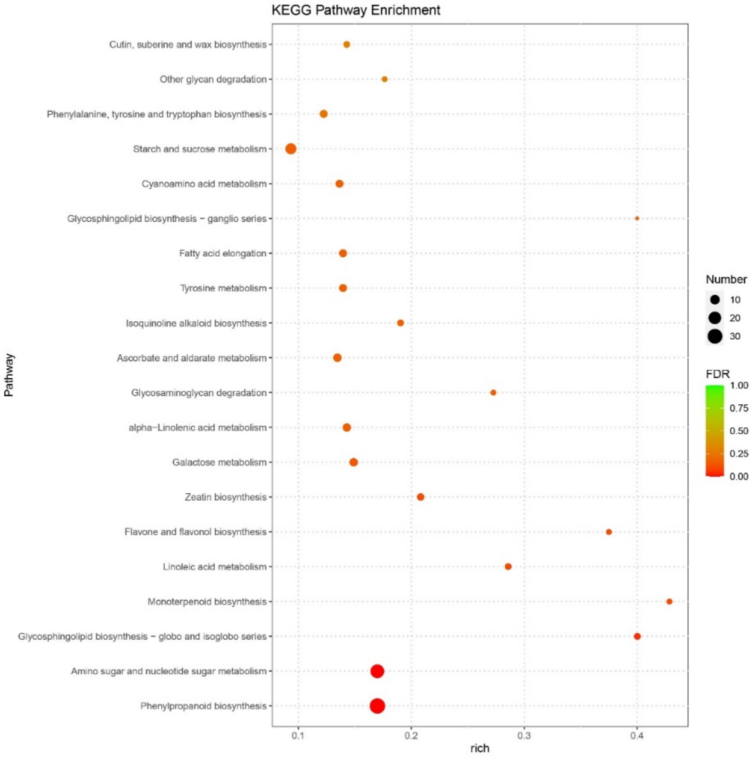

Figure S2. Statistics of pathway enrichment analysis of differential expression genes between wild-type (WT) and *yld7* plants. mRNA was purified from total RNA isolated from tillering-stage plants of WT('changchungu') and *yld7*.

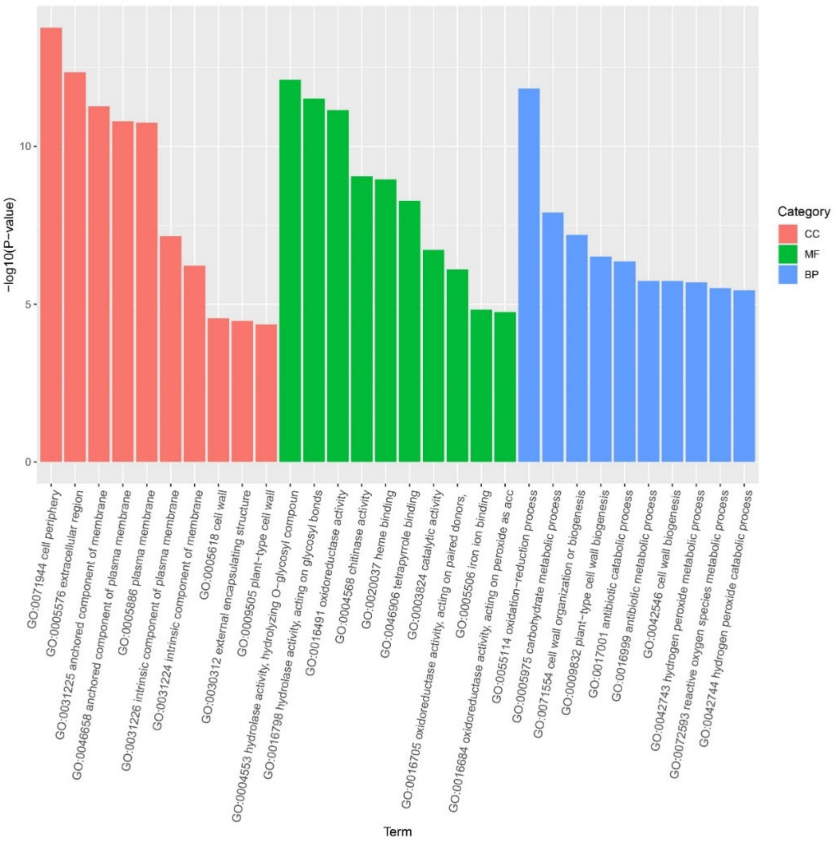

Figure S3. The number of differential expression genes involved in different biological processes, cellular， components and molecular functions. mRNA was purified from total RNA isolated from tillering-stage plants of WT(‘changchungu’) and *yld7*.
